# Climate-Based Models for Pulsed Resources Improve Predictability of Consumer Population Dynamics: Outbreaks of House Mice in Forest Ecosystems

**DOI:** 10.1371/journal.pone.0119139

**Published:** 2015-03-18

**Authors:** E. Penelope Holland, Alex James, Wendy A. Ruscoe, Roger P. Pech, Andrea E. Byrom

**Affiliations:** 1 Landcare Research, Lincoln, Canterbury, New Zealand; 2 Department of Biology, University of York, Heslington, York, United Kingdom; 3 Biomathematics Research Centre, University of Canterbury, Christchurch, New Zealand; 4 Institute for Applied Ecology, University of Canberra, Bruce, ACT, Australia; U.S. Geological Survey, UNITED STATES

## Abstract

Accurate predictions of the timing and magnitude of consumer responses to episodic seeding events (masts) are important for understanding ecosystem dynamics and for managing outbreaks of invasive species generated by masts. While models relating consumer populations to resource fluctuations have been developed successfully for a range of natural and modified ecosystems, a critical gap that needs addressing is better prediction of resource pulses. A recent model used change in summer temperature from one year to the next (ΔT) for predicting masts for forest and grassland plants in New Zealand. We extend this climate-based method in the framework of a model for consumer–resource dynamics to predict invasive house mouse (*Mus musculus*) outbreaks in forest ecosystems. Compared with previous mast models based on absolute temperature, the ΔT method for predicting masts resulted in an improved model for mouse population dynamics. There was also a threshold effect of ΔT on the likelihood of an outbreak occurring. The improved climate-based method for predicting resource pulses and consumer responses provides a straightforward rule of thumb for determining, with one year’s advance warning, whether management intervention might be required in invaded ecosystems. The approach could be applied to consumer–resource systems worldwide where climatic variables are used to model the size and duration of resource pulses, and may have particular relevance for ecosystems where global change scenarios predict increased variability in climatic events.

## Introduction

Mast seeding (synchronous intermittent production of large seed crops [1]) is a major driver of the abundance and dynamics of small mammal and bird consumers [2–5]. Resource pulses such as mast seeding can influence dynamics and interactions among species at multiple trophic levels [2, 6, 7] and, for some ecosystems, accurate predictions of consumer responses to pulsed resources are critical for managing unwanted impacts at one or more trophic levels (e.g. [8]). Where mast events drive the dynamics of pest species, low-cost predictions of pest outbreaks can be critical for maximizing the benefit-to-cost ratio of control programmes [9].

Prevention of damage by outbreaking consumer species often requires management to be implemented or budgeted for well in advance of resource pulses [10]. In addition to control of invasive species, accurate forecasting is required for managing disease and parasite outbreaks that threaten wildlife [11], mitigating the impacts of zoonoses [12] and, in agricultural systems, preventing plagues of crop or pasture pests [13–15]. This can be achieved by (i) predicting the likely size and occurrence of resource pulses and (ii) estimating the response of consumer populations to those resources. The latter problem is more easily overcome than the former: given appropriate long-term field data, statistical and mechanistic models relating consumer populations to resource fluctuations have been successfully developed for a range of natural and modified ecosystems [2, 16, 17].

The critical gap that now needs addressing is better prediction of the size and occurrence of resource pulses, which requires good understanding of the influence of key drivers such as climate, in particular temperature and rainfall. While temperature is known to be a driver of masts [18, 19], temperature cues are often modelled in combination with other predictors such as rainfall or nutrient availability [19–24]. Although predictive power can be improved by including several variables, these are often site-specific and do not have the ‘one-size-fits-all’ advantage of using a single generic predictor.

Kelly et al. [25] showed that change in average summer temperature from one year to the next (ΔT, 1 and 2 years prior to seed production) is a significantly better predictor of masts than absolute temperature (T) for a range of genera in New Zealand (improving predictions for mast seeding by about 50% on average compared with previously published work; see Kelly et al. [25]). Using the ΔT model reduces the odds of failing to predict a mast year (a false negative), or expecting high seedfall when it is not warranted (a false positive). These scenarios have significant ramifications for the management of invasive consumer species, since a false negative may result in high levels of damage, whereas too many false positives could undermine planning and budgeting. Although Kelly et al. [25] noted that predictions could be improved for some species and sites, for example by fine-tuning the section of summer months or adding environmental covariates, the relatively minor gains from this process detract from the benefits of a simple, universal ΔT model for species of management interest.

The primary focus of this paper is to predict consumer outbreaks using improved climate-driven predictions for resource pulses. The ultimate goal is to predict consumer responses directly using climate data without having to collect resource data or monitor consumers directly on an annual basis, as climate data are inexpensive to obtain across large areas, and a climate-based prediction might provide additional time to plan and implement management to prevent potential outbreaks. An appropriate climate driver is therefore key. We develop a simple, mechanistic consumer–resource model including production and depletion of resources and demographic responses of the consumer, and explore how different climate drivers for pulsed resources affect the magnitude and occurrence of masts, and their influence on consumer abundance. The model is designed for mouse population dynamics determined primarily by resource inputs [16]: environmental factors determine food availability and therefore the rate of increase of mice, subject to relatively minor density-dependent mechanisms. Food consumption by mice contributes to the decline in food supply, which is also affected by factors unrelated to mouse abundance. To parameterize the model, we use data on temperature, hard beech (*Fuscospora truncata*) seedfall, and invasive house mouse (*Mus musculus*) abundance from the Orongorongo Valley (OV), New Zealand. Mice respond strongly to beech seed availability [16, 26], but are eaten by invasive stoats (*Mustela erminea*), prompting an increase in stoat abundance. Predation by both mice and stoats results in reduced survival of invertebrates [27] and native birds [26, 28, 29]; hence the need for timely intervention to protect vulnerable native fauna. We fitted the consumer–resource model to the OV mouse data using observed seedfall, and seedfall predicted by the T and ΔT [25] models. We demonstrate that the choice of climate driver can have a significant influence on the explanatory and predictive power of the consumer–resource model.

## Methods

### Overview

Model development followed a sequence of four steps. First, we describe the field data. Second, we use these data to parameterize and compare a set of consumer–resource models. Third, we describe two methods for predicting annual seedfall from summer temperature data and use these to re-estimate parameters for the best consumer–resource models. Fourth, we use observed seedfall and mouse abundance, and the output of the best temperature-based seedfall model in the best consumer–resource model, to develop simple, robust relationships for predicting increases in mouse abundance during periods of beech seed availability, and the annual peak in mouse abundance.

#### Step 1: Field data

Data on temperature, seedfall and mouse abundance were collated for mixed beech–podocarp–broadleaved forest in OV (New Zealand’s North Island: 41° 21′ S, 174° 58′ E). For 43 years (1968–2010), seed production (seeds m^−2^) by hard beech was measured using permanent seed trays mounted *c*. 1.5 m above the forest floor [19]. Seedfall from beech trees occurs primarily in late summer and autumn, i.e. February to May [30]. Mean summer temperature was calculated from mean monthly air temperatures (January–March), measured at the nearest weather station (Kelburn). An index of mouse abundance (captures per 100 trap nights; C/100TN) was measured quarterly for the 25 years from May 1972 to November 1996. Trapping sessions were conducted in Austral summer (February), autumn (May), winter (August) and spring (November) using 116 kill-traps spaced at 50-m intervals along a 4-km transect running through the forest parallel to the hill slope at 100–200 m elevation [31]. Data were used from the 30% of these traps located in predominantly beech forest, transformed to adjust for trap saturation [16]. To fill in data for a full initial year, mouse abundance in February 1972 was assumed to be equal to the minimum February value of the time series (1·0 C/100TN). The start of each annual cycle was taken to be February. Temperature, seedfall and mouse trap-catch data are available at http://dx.doi.org/10.7931/J2W66HPB.

#### Step 2: Consumer–resource models

The rate of change of the mouse population *M* was assumed to depend on food consumption [32] and consumer density [33]:

$$\frac{dM}{dt}=\left(\alpha g(F)-\mu_{1}-\mu_{2}M\right)M.$$ Eqn 1

For all equations, time *t* is continuous and time-dependent parameters are measured in units of years. Mouse abundance is quantified by the index C/100TN, *F* is food availability (seeds m^−2^), *g*(*F*) is the functional response (per capita consumption of seed by mice) and α is the demographic efficiency of mice, i.e. their ability to convert food into recruitment for the mouse population. The parameters μ_1_ (year^−1^) and μ_2_ (mouse^−1^ year^−1^) are density-independent and density-dependent rates respectively [33]. Both μ_1_ and μ_2_ may be positive or negative, depending on non-food-related processes (e.g. predation, social interactions, Allee effects, etc.).

Four candidate models for *g(F)* were tested:

- Holling I (piecewise linear): *g(F)* = *c*_*1*_ min(*F*, *K*)
- Holling II (Ivlev): *g(F)* = *c*_*2*_ (1—exp(—*e F*))
- Holling II (standard): *g(F)* = *c*_*2*_ *F* / (*F*+*K*)
- Uncapped: *g(F)* = *c*_*1*_ *F*

In all four models, when food biomass is low, per capita consumption increases with the amount of food available. Model A is a type I functional response and models B and C are variations of a type II functional response: when food biomass is high, consumption slows due to constraints on search and handling time to reach a maximum rate that is capped either at a single value (when *F* exceeds *K* seeds m^−2^ in model A) or an asymptote (models B and C). Model D is uncapped and the per capita intake rate will continue to increase at high food levels. In model B, *e* ((seeds m^−2^)^–1^) is a measure of foraging efficiency. In model C, *K* (seeds m^−2^) is the food availability at which growth is at half the possible maximum rate, in the absence of all other effects. In models A and D, *c*_*1*_ (mouse^−1^ year^−1^) is the feeding rate of mice, and in models B and C, *c*_*2*_ (seeds m^−2^ mouse^−1^ year^−1^) is the maximum per capita feeding rate. There are many other models available for the functional response (e.g. [34]); these were chosen for their simplicity and to minimise the number of parameters to be estimated.

The rate of change of available food was modelled as:

$$\frac{dF}{dt}=S\left(t\right)-hF-g\left(F\right)M.$$ Eqn 2

The first term, *S*(*t*), describes changes in available food, modelled in the *y* ^th^ annual cycle as a total amount *F*_*y*_ delivered at a constant rate over the first quarter of the year:

$$S\left(t\right)=\{\begin{array}{c} \frac{F_{y}}{0.25}\;\text{if}\;0>\text{floor}\left(t\right)>0.25;\\ 0\;\text{otherwise}. \end{array}$$ Eqn 3

The floor function, which rounds *t* down to the largest integer smaller than *t*, allows *S*(*t*) to increase during autumn only (February–April of each year). The second term, *hF*, describes the change in available food that happens throughout the year at a constant rate *h* (year^−1^) unrelated to mouse abundance. The third term, *g(F) M*, describes the rate of seed consumption by mice. Food was not carried over between years; it was reset to zero at the start of each annual cycle.

We took the annual values of *F*_*y*_ to be the observed annual seedfall from the OV data, starting from February 1972. We modelled the abundance of mice over the next 25 years with Eqns 1–3 and with each candidate functional response. Model time is continuous but the data are from discrete points in time. The abundance of mice at the start of each quarter was therefore extracted to be compared with the data. Best-fit parameter values were chosen by comparing the predicted seasonal mouse abundances with those for OV mouse abundance (quarterly, February 1972 to November 1996; number of data points = 100), and minimising the root mean square error (RMSE). Confidence intervals were estimated from the distribution of 100 best-fit values found using the non-parametric bootstrapping technique of sampling with replacement [35]. Best-fit parameter values were used to generate predicted mouse abundance and candidate consumer models were compared using AIC_c_ [35] and Pearson’s *r* correlation. Even though data were collected over 25 years, only five large seedfall events (log_10_(seedfall) > 1) and seven smaller seedfall events (0 < log_10_ (seedfall) < 1) occurred during the period, and there were seven periods for which mouse abundance was high (above 2.0C/100TN). It was therefore not feasible to test model fit using techniques such as splitting the data set and assessing the predictive power of a model trained on a reduced data set.

We tested each formulation of the consumer–resource model with a reduced number of parameters corresponding to minimal effects of processes such as predation or intraspecific competition for mice, or loss of seed to germination or decay (i.e. with either μ_2_ = 0 or μ_1_ = 0, and/or *h* = 0). The best models were chosen for their low AIC_c_ value, and these models were used for all subsequent analyses.

#### Step 3: Predicted seedfall

To predict mouse abundance from climate data, we first required a way of predicting seedfall. Kelly *et al*. [25] showed that seedfall *F*_*y*_ (seeds m^−2^) could be modelled using the mean summer temperature (daily average for the 3-month period January to March) in the previous year (*T*_*y–1*_) or the previous 2 years (*T*_*y–1*_ *− T*_*y–2*_). We tested both seedfall models as drivers for the consumer–resource model.

T model: We used linear regression to predict *F*_*y*_ as a function of temperature in the previous summer, fitting log_10_(*F*_*y*_) = *a + b T*_*y–1*_ to the OV beech seedfall data over the period for which the index of mouse abundance was measured (1972–1996).

ΔT model: We used linear regression to predict *F*_*y*_ as a function of the change in temperature in the previous two summers, fitting log_10_(*F*_*y*_) = *a + b*(*T*_*y–1*_ *− T*_*y–2*_) to the OV beech seedfall data (1972–1996).

We tested each seedfall model for autocorrelation, and compared the distributions of seedfall data produced by each model to each other and to the observed OV seedfall using a Kolmogorov–Smirnov test.

Parameters for the best consumer–resource models from Step 2 were re-estimated by replacing the values of *F*_*y*_ in Eqn 3 previously taken from the field data with values predicted from either the T model or the ΔT model, based on temperatures for 1972–1996 for OV. Confidence intervals for these parameter values were found using bootstrapping methods as above. AIC_c_ and Pearson’s *r* correlation were used for comparing models.

#### Step 4: Annual predictions of the increase and peak abundance of mice

We generated a 1000-year time series of mean summer temperatures by sampling from a normal distribution fitted to the OV mean summer temperature values. The validity of this approach was tested by Holland and James [36], who showed that the temperature time series from the OV (among others) was normally distributed and showed no autocorrelation on the first five lags, and that the data sampled in a random order, compared with the true order, had the same distribution of times between years with temperature in the top 25%, and the same frequency of events where either high summer temperatures or high positive values of ΔT predicted masts in two consecutive years. This 1000-year time series was used to generate a seedfall time series using the ΔT model, and a corresponding time series for mouse abundance using the Ivlev model B with the parameter-set based on observed seedfall (results were comparable for models A and C with corresponding best-fit parameter sets). The length of this data set was chosen to capture a large number of single and double mast events over the course of the simulation, despite their rarity. We extracted quarterly values of mouse abundance from the continuous model output, to compare with field data, and calculated the finite rate of increase during the period from late summer to early spring (mid-February to mid-August), i.e. from immediately prior to annual production of beech seed to the post-seedfall quarter when mouse populations approach peak annual abundance [17]. We plotted four cases: the 6-month increase and the early spring (August) value of the index for each year against the corresponding ΔT value (from the previous 2 years) and modelled seedfall in the preceding autumn. In all four cases the model output was used to compare logistic and linear relationships between mouse demographics and ΔT or seedfall. The goodness of fit for the possible relationships was compared using AIC_c_ and Pearson’s *r* correlation (*r*_*mm*_). The observed OV data were treated in the same way as the model output, i.e. the increase of mice from late summer to early spring, and the spring value of mouse abundance, were plotted against the appropriate ΔT and seedfall values. Again the goodness of fit of linear and logistic relationships was compared with AIC_c_ and Pearson’s *r* correlation (*r*_*dd*_). Finally, model predictions and the data were compared in all four cases using the fitted logistic curves and the data (*r*_*dm*_).

### Effect of climate change on resource pulses driving consumer outbreaks

Following Tompkins *et al*. [37], we defined a mast year as one in which seed production was greater than the long-term mean by a designated amount based on standard deviates (Method 4 in [38]). We calculated the annual deviate from the long-term mean, *AD*_*y*_, from the 43 years of OV seedfall data (*F*_*y*_) as

$$AD_{y}=\frac{F_{y}-\text{mean}\left(F_{y}\right)}{\text{standard deviation of}\;F_{y}}$$ Eqn 4

Years in which *AD*_*y*_ was greater than a threshold *AD*_thres_ = minimum(absolute value(*AD*_*y*_)) were considered to have a relative high, positive standardized deviate, and can be called mast events. We calculated the minimum temperature (T_thres_) and minimum temperature change (ΔT_thres_) for the mast years identified in the OV data.

Predictions of annual mean summer temperature (January–March) at OV from 1972 to 2100 were generated by the National Institute of Water and Atmospheric Research [39] for three climate scenarios: A2 (which corresponds to ‘regionally oriented economic development’), B1 (which corresponds to ‘global environmental sustainability’) and the intermediate case, A1B [40]. A systematic bias was evident with lower mean and variance of temperatures in all climate scenarios compared with the overlapping years of climate scenarios and historical OV temperature data, likely resulting from the scenarios having been interpolated for a 5-km-grid cell. We adjusted the mean and variance of the climate scenarios to match the mean and variance of the OV temperature data using the overlapping years 1972–2010, to account for this local variation. We then calculated ΔT time series from the adjusted climate scenario data. For the 43 years of observed ΔT values from the OV, and for each 100-year T and ΔT time series for the three adjusted climate scenarios, we calculated the proportion of years in which a single mast event should occur (i.e. *T*_*y*_ ≥ T_thres_ or Δ*T*_*y*_ ≥ ΔT _thres_), the proportion of years in which the first year of a double mast event (i.e. two consecutive mast events) should occur (i.e. *T*_*y*_ & *T*_*y*+1_ ≥ T_thres_ or Δ*T*_*y*_ & Δ*T*_*y*+1_ ≥ ΔT _thres_), and the average time between single mast events (i.e. *t* for which *T*_*y*_ & *T*_*y*+t_ ≥ T_thres_ and *T*_*k*_ < T_thres_, or Δ*T*_*y*_ & Δ*T*_*y*+t_ ≥ ΔT_thres_ and Δ*T*_*k*_ < ΔT_thres_, for *y+1* < *t* < *k*).

## Results

### Model selection

AIC_c_, RMSE and correlation (Pearson’s *r*) values for the various candidate models fitted to the OV mouse abundance data are shown in Table 1. In all cases μ_2_ (density-dependent mortality) and *h* (external seed loss) were included in the model; without these terms the AIC_c_ values increased significantly (ΔAIC_c_ > 20). Initially the model was highly unstable to parameter fitting, in all cases tending to give very small values for *c*_*1*_ and *c*_*2*_, and very wide confidence intervals for α. This was corrected by removing the third term from Eqn 2 (meaning that feeding by consumers did not have a significant effect on food availability) and combining the *c* and α parameters in Eqn 1 (i.e. setting α = 1). This model simplification gave an improved fit, as measured by RMSE and AIC_c_ and narrower confidence intervals for the remaining parameters. All three capped models with the model simplification and α = 1 gave an equally good description of the mouse abundance data (AIC_c_ values differed by <1), and all three had Pearson’s correlation *r* = 0·74 and RMSE = 1·45.

**Table 1:** Comparison of Four Consumer–Resource Models Fitted to Mouse Abundance Data from the Orongorongo Valley, New Zealand.

*g(F)*	Density-dependent term	Density-independent term	Other food changes	*N*	RMSE	Pearson’s *r*	ΔAIC_C_
**A—Piecewise**	−*μ*_*2*_ *M*		−*hF*	4	1·53	0·73	19·7
	**−*μ***_***2***_ ***M***	**−*μ***_***1***_	**−*hF***	**5**	**1**·**45**	**0**·**74**	**0**
**B—Ivlev**	−*μ*_*2*_ *M*		−*hF*	4	1·52	0·70	16·2
	**−*μ***_***2***_ ***M***	**−*μ***_***1***_	**−*hF***	**5**	**1**·**45**	**0**·**74**	**0**·**23**
**C—Holling II**	−*μ*_*2*_ *M*		−*hF*	4	1·50	0·72	9·3
	**−*μ***_***2***_ ***M***	**−*μ***_***1***_	**−*hF***	**5**	**1**·**46**	**0**·**74**	**0**·**52**
**D—Uncapped**	−*μ*_*2*_ *M*		−*hF*	3	1·78	0·29	78·6
	−*μ*_*2*_ *M*	−*μ*_*1*_	−*hF*	4	1·65	0·50	50·3

Observed seedfall (*F*) was used as input for the models. The models included different combinations of functional response (*g(F)*), density dependence (*μ*_*2*_ *M*) and density independence (*μ*_*1*_) in the rate of increase for mice, and changes in food (*hF*) unrelated to mouse abundance. All models shown excluded the functional response term from the resource equation (Eqn 2) and had fixed α = 1. The models had *N* parameters (not including α) and comparisons were based on root mean square error (RMSE), Pearson’s *r* correlation and the corrected AIC_C_. Models with lowest AIC_C_ values are shown in bold.

The best-fit parameter values and associated confidence intervals for the three best models using the OV seedfall data are shown in Table 2. The predicted parameter values and confidence intervals were very similar for all three models. However, while the confidence intervals were relatively stable for the observed seedfall and ΔT models (i.e. when bootstrapping was repeated, the same values were generated to two decimal places), values for the T model were more variable. The linear term in the rate of change of the mouse population was negative in all cases (μ_1_ ≈ −1·2), and the density-dependent term for all models was positive (μ_2_ ≈ 0 75), suggesting regulation by processes not related directly to seed availability. Together these terms suggest that in the absence of beech seedfall effects, expected mouse abundance in OV would be approximately 1·6 (= −μ_1_/μ_2_) C/100TN. All models predicted the seed-loss term to be *h ≈* 9. In the absence of mouse predation this corresponds to a seed half-life of approximately 6 weeks and depletion of the seed bank to <5% in 6 months, which is consistent with observations of germination [30].

**Table 2:** Parameter Values for the Best-Fit Consumer Models Fitted to Mouse Abundance.

Consumer Model	A—Piecewise		B—Ivlev		C—Standard	
**Seedfall—Observed**						
*c*_1_ (seeds mouse^−1^ year^−1^) or *c*_2_ (mouse^−1^ year^−1^)	4·41	(1·5, 17·8)	6·74	(5·3, 10·2)	6·95	(5·5, 10·6)
*K* (seeds) or *e* (seeds^−1^)	1·49	(0·4, 3·9)	1·08	(0·3, 4·4)	0·67	(0·08, 4·0)
μ_1_ (year^−1^)	−1·31	(−2·0, −0·6)	−1·23	(−2·1, −0·4)	−1.05	(−2·3, −0·2)
μ_2_ (mouse^−1^ year^−1^)	0·77	(0·5, 1·1)	0·76	(0·5, 1·3)	0.71	(0·5, 1·2)
*h* (year^−1^)	9·18	(5·1, 16.1)	9·48	(4·8, 18·5)	9.80	(4·8, 17·1)
Correlation	0·74		0·74		0·74	
**Seedfall Model—Δ*T***						
*c*_1_ (seeds mouse^−1^ year^−1^) or *c*_2_ (mouse^−1^ year^−1^)	5·36	(1·6, 7·8)	7·15	(5·1, 11·8)	7·80	(3·4, 27·2)
*K* (seeds) or *e* (seeds^−1^)	1·36	(0·9, 3·5)	1·25	(0·5, 10·2)	2·29	(0·5, 21·3)
μ_1_ (year^−1^)	−0·99	(−2·5, −0·5)	−0·75	(−1·9, 0·5)	−3·55	(−8·4, −0·9)
μ_2_ (mouse^−1^ year^−1^)	1·00	(0·5, 1·8)	0·87	(0·5, 1·7)	2·13	(0·8, 7·2)
*h* (year^−1^)	4·47	(1·9, 8·8)	5·38	(2·2, 16·1)	3·32	(−0·7, 13·4)
Correlation	0·71		0·71		0·71	
**Seedfall Model—T**						
*c*_1_ (seeds mouse^−1^ year^−1^) or *c*_2_ (mouse^−1^ year^−1^)	4·39	(2·1, 85·3)	7·87	(3·4, 27·2)	11v57	(4·4, 25·5)
*K* (seeds) or *e* (seeds^−1^)	0·76	(0·3, 2·9)	2·29	(0·5, 21·3)	0·085	(0·0, 0·8)
μ_1_ (year^−1^)	−1·76	(−46·5,−1·1)	−3·55	(−8·4, −0·9)	−0·19	(−4·5, 0·2)
μ_2_ (mouse^−1^ year^−1^)	0·86	(0·7, 33·7)	2·13	(0·8, 7·2)	2·17	(0·8, 6·1)
*h* (year^−1^)	4·44	(0·00, 11·9)	3·32	(−0·7, 13·4)	3·9	(0·1, 19·3)
Correlation	0·42		0·41		0·41	

Parameter values for the best-fit consumer models fitted to mouse abundance using observed seedfall or seedfall predicted by the change in mean summer temperature in the preceding 2 years (ΔT) or mean summer temperature last year (T). Model parameters are defined in the text. Values in brackets are 95% confidence intervals. Correlation between the consumer model predictions and field-collected data on mouse abundance was measured by Pearson’s *r*. The index of mouse abundance is captures per 100 trap nights, ‘seeds’ is shorthand for ‘seeds m^−2^’.

### Predicting resource fluctuations

The ΔT model (log_10_(*F*_*y*_) = 0·33 *+* 0·97(*T*_*y–1*_ *− T*_*y–2*_), *p* < 10^−7^, *r* ^2^ = 0·53) was a better predictor of historical seed resources than T (log_10_(*F*_*y*_) = −18·61 *+* 1·14 *T*_*y–1*_, *p* < 10^−4^, *r* ^2^ = 0·35). The distribution of seedfall given by the data, and the T and ΔT predictors, showed no significant differences (Kolmogorov–Smirnov test, statistic < 0·001). The observed OV seedfall time series, and those from the T and ΔT predictors, showed no evidence of autocorrelation (Ljung–Box Q-test, *P* > 0·05 for the first three lags in all series).

### Modelling consumer abundance using temperature to predict seedfall

The best-fit model parameters with confidence intervals and correlation coefficients for the three best models using temperature-based seedfall drivers are shown in Table 2. Recall that for these models, the OV temperature time series was used to model seedfall, and then mouse abundance, and the model output was compared with OV mouse abundance data. Best-fit model parameters using ΔT were within the 95% confidence interval of the corresponding best-fit parameters using observed seedfall, although the ΔT driver predicted a slower rate of seed decay.

As measured by Pearson’s *r* correlation, the ΔT-driven model was as good a predictor of mouse abundance as the observed seedfall (ΔT: *r* = 0.71; observed seedfall: *r* = 0.74), but the T-driven model was a substantially worse predictor (T: *r* = 0.41).

**Fig 1:**
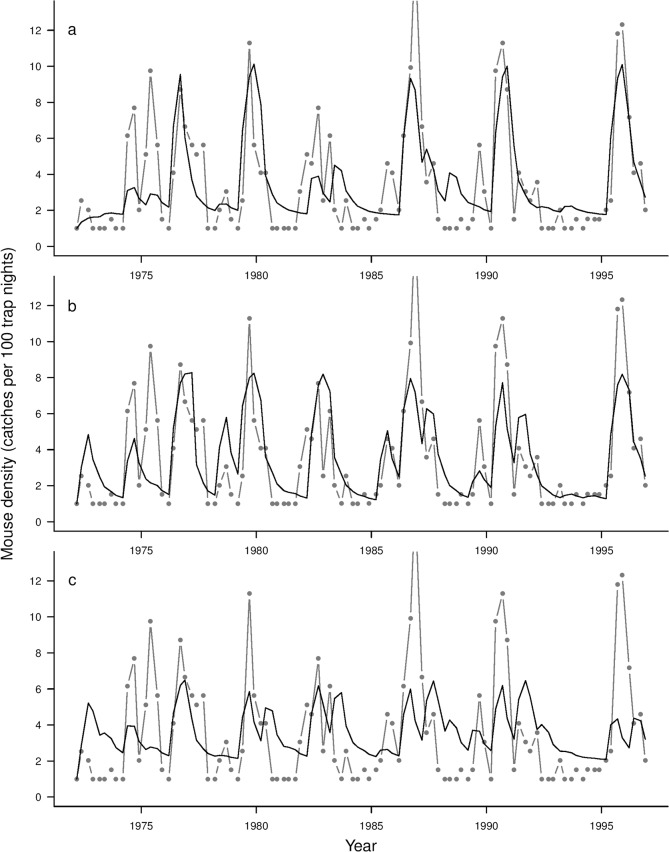
Quarterly abundance of mice, Orongorongo Valley, New Zealand. Abundance (grey points/lines) and the predicted time series from a consumer model (solid dark line) using the best-fit parameter values and the Ivlev model fitted using three seedfall drivers: (a) observed seedfall; (b) seedfall predicted using ΔT (change in mean summer temperature in the preceding 2 years) and (c) seedfall predicted using absolute temperature T (mean summer temperature last year). Model parameters are in Table 2.

The predicted mouse abundance using best-fit parameter values for the consumer model with an Ivlev functional response driven by observed seedfall, and seedfall derived using the ΔT or T predictors, is shown in Fig. 1. None of the models fully captured the observed magnitude of the largest mouse outbreaks, but models using observed seedfall, or seedfall predicted using ΔT, did predict large outbreaks in those years.

### Predicting consumer outbreaks

**Fig 2:**
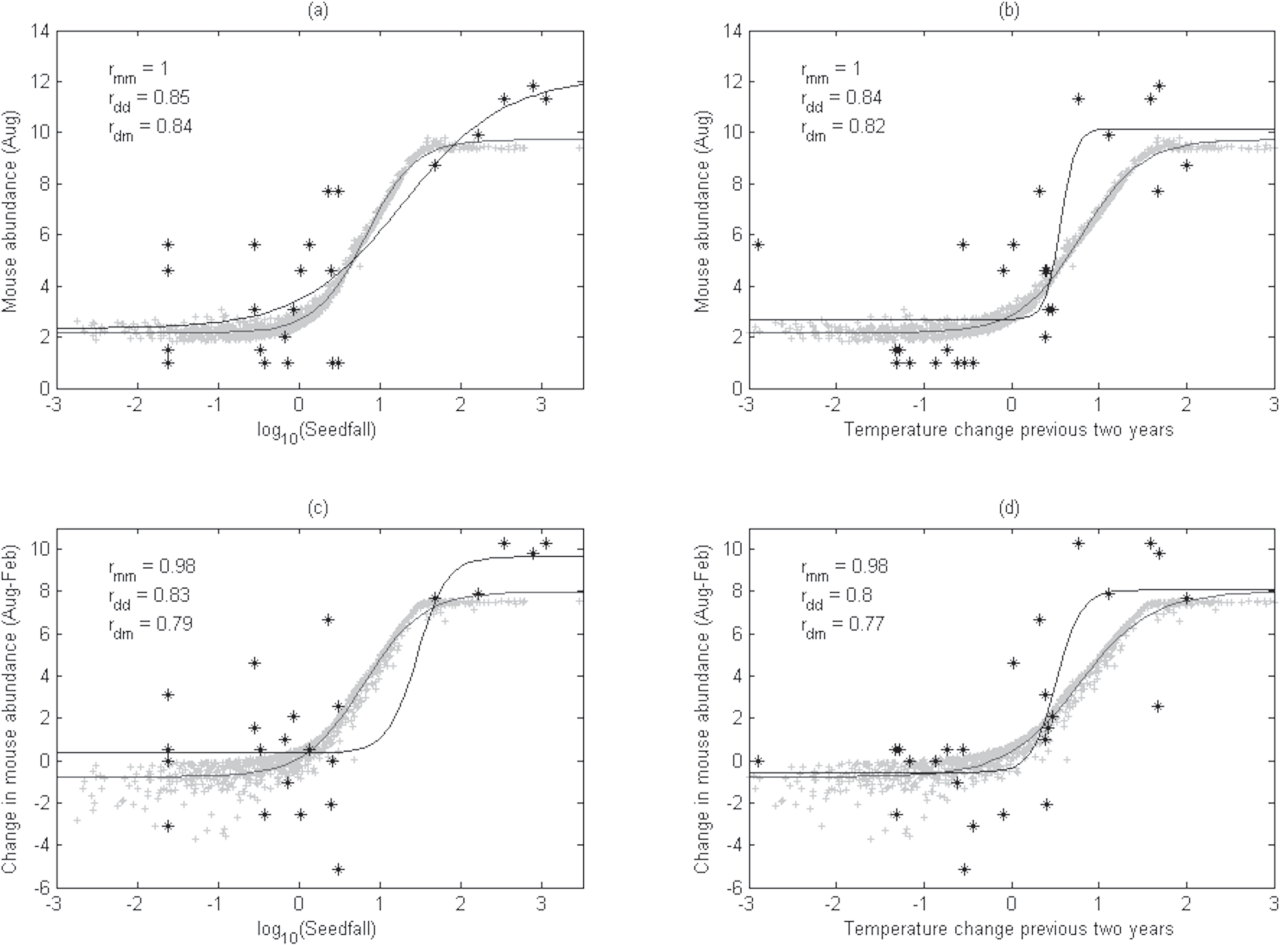
Relationships predicting the annual spring peak in abundance and changes in OV mouse population over autumn/winter. Mouse abundance (C/100TN) in early spring (August) and change in mouse abundance from late summer to early spring (February–August) are plotted against (a, c) log_10_(seedfall) from the preceding year and (b, d) the mean summer temperature change between the two previous years (ΔT). In all panels, modelled results and the best-fit logistic curve are shown in grey. Observed demographic data and the best-fit logistic curve are shown in black. Correlation values are *r*_*mm*_ between the model (grey) line and the model (grey) points, *r*_*dd*_ between the data (black) line and the data (black) points, and *r*_*dm*_ between the model (grey) line and the data (black) points.

A logistic curve gave a better fit than a linear regression when plotting the change in mouse abundance from late summer to early spring or peak abundance in spring (calculated from the 1000-year simulated time series) against either ΔT (from the previous 2 years) or autumn seedfall (Fig. 2). This is in accordance with mouse populations rapidly achieving their maximum rate of increase during mast years in New Zealand ecosystems (e.g. [17]). In all four cases the improvement in fit given by the logistic curve was large (ΔAIC > 10). The correlation between the model output and the fitted logistic curve was very high (Pearson’s *r*: *r*_*mm*_ > 0·98) in all cases.

Similarly, when OV field data on spring mouse abundance and the change in mouse abundance from late summer to early spring were plotted in the same way, in all four cases a logistic curve gave a better fit than a linear regression, as measured by both AIC and correlation coefficient (Fig. 2). In three of the four cases the improvement was moderately large (ΔAIC_c_ > 5). However, for the data there was one case (mouse abundance predicted by log_10_(seedfall)) where the improvement with the logistic model was only marginal (ΔAIC_c_ < 2). The correlation between the data and the best-fit logistic curve was high (Pearson’s *r*: *r*_*dd*_ > 0·8) in all cases.

The strongest correlations for the model and data were between the observed mouse abundance in spring and either observed seedfall (*r*_*dd*_ = 0·85, *r*_*mm*_ > 0·99) or ΔT (*r*_*dd*_ = 0·84, *r*_*mm*_ > 0·99) (Fig. 2A-B).

Finally, correlation between the model output (i.e. the logistic curve fitted to the simulation data) and the observed data from OV (*r*_*dm*_) was also strong in all four cases. Again, the strongest correlations were between the simulated mouse abundance in spring and either observed seedfall (*r*_*dm*_ = 0·84) or ΔT (*r*_*dm*_ = 0·82) (Fig. 2A- B).

Logistic curves fitted to mouse abundance in spring against observed seedfall had a relatively shallow gradient (i.e. a linear regression was only a slightly worse fit) for both model and data (Fig. 2A). A data gap around log_10_(seedfall) = 1 (likely resulting from the on–off nature of beech seedfall) makes identifying a clear threshold difficult. In contrast, ΔT values do not show a clear gap (i.e. all values of ΔT are equally likely to occur, and the logistic curve fitted to the observed mouse abundance in spring against ΔT had a very steep slope (Fig. 2B), suggesting a clear threshold). A threshold was also evident when the change in mouse abundance from late summer to early spring was plotted against ΔT (Fig. 2D). For ΔT > 1°C, the change in mouse abundance and the observed peak abundance in spring were high. In contrast for ΔT < 0°C both these values were low.

### Effect of climate change on resource pulses driving consumer outbreaks

Eight years of seedfall data from the OV were designated as mast years (0.19 of years, every 5.43 years on average; Table 3). The minimum value of ΔT in these 8 years was ΔT_thres_ = 0.77°C, which is consistent with the ΔT thresholds for change in mouse abundance and peak abundance identified in the previous section. The corresponding minimum value of T was T_thres_ = 16.67°C. Using ΔT_thres_ to predict mast events from three climate scenarios over the period 2001–2100 suggests slightly more frequent mast events (ranging from 21% to 24% of years, every 4.20–4.32 years on average), with only two double mast events occurring during the century (Table 3). In contrast, 83–92% of years are predicted to be above T_thres_, with consecutive mast events being the norm rather than the exception (Table 3). This is due to the increasing trend in temperatures in the climate scenarios: four of the first 10 years are predicted to have T ≥ T_thres_, but all 10 years of the final decade of the century have T ≥ T_thres_. For the ΔT time series, one year in the first decade and two years in the final decade have ΔT ≥ ΔT_thres_.

**Table 3:** Effect of climate change on mast events.

	Historical Data	Climate Scenario		
	Observed seedfall	A2	A1B	B1
**T Model**				
Single mast events	0.19	0.89	0.92	0.83
Double mast events	0	1.13	1.08	1.21
Average yrs between mast events	5.43	1.86	1.98	1.58
**ΔT Model**				
Single mast events	0.19	0.23	0.24	0.21
Double mast events	0	0.02	0.02	0.02
Average yrs between mast events	5.43	4.32	4.26	4.20

Proportion of years in which there is expected to be a single mast event; proportion of years in which the first year of two consecutive mast events may occur (double mast events); and the average time between single mast events, calculated from observed seedfall data, and predicted from simulated 100-year time series for three climate scenarios.

## Discussion

Predicting the occurrence and magnitude of resource pulses as far in advance as possible is fundamental to predicting, budgeting for and managing outbreaks of consumers and for understanding cascading consequences among multiple trophic levels [7]. In particular, predictions based on low-cost, readily available data, such as climatic data, can overcome the financial and logistic constraints of field-based methods for monitoring either resources or consumers [9]. Predicting longer-term changes in the frequency of consumer outbreaks is also critical for adjusting management strategies in natural and human-modified ecosystems [37, 41, 42]. Our aim was to improve models for pulsed resources, in order to improve the predictability of consumer outbreaks. We found that, for a model system of invasive house mice (consumers) in New Zealand beech forest, an improved climate-based model for pulsed resources (the ΔT model [25]) resulted in more accurate predictions for fluctuations in consumer abundance. In addition, the ΔT-driven consumer model displayed threshold effects, supporting the use of even stronger thresholds between mouse demographic data and ΔT to indicate when outbreaks may occur, and hence improve the timing of management interventions. These direct forecasts of mouse outbreaks, rather than inferred outbreaks from predictions of masts, are required for optimal management of invasive mammals that threaten indigenous fauna in beech forests [9].

Models of seed production using differential temperature cues appear to be applicable to a wide range of plant genera in New Zealand [25], North America [23, 43], and Europe [44], although other cues may also be involved [22, 23, 45]. Masts can have cascading trophic effects on ecosystems by increasing the abundance of top predators [46], which may in turn reduce the abundance and productivity of secondary prey [4, 28, 47]. Resource pulses also influence omnivores [48] and herbivores [12], disease outbreaks [12, 49], parasite prevalence [50, 51] and invertebrate abundance [12, 20, 21]. Hence, our findings have general applicability for a range of natural and human-modified ecosystems worldwide.

Our model parameters and outputs were reassuringly similar to previous models of the population dynamics of invasive house mice. For instance, the best consumer model had a negative density-dependent growth term and a positive independent growth term, like that of Choquenot and Ruscoe [16]. Our best models had a capped functional response to food availability, consistent with previous field observations of satiation in seed consumption by mice [17]. Our modelled maximum finite rate of increase over 6 months was a little less than the observed value *≈*10 (Fig. 2C- D), similar to the maximum instantaneous rate of increase of 0·5 per 40 days estimated by Pech et al. [52] for house mouse outbreaks in Australia. Further, our estimates of the half-life of beech seed available using the ΔT driver were 12–16 weeks: consistent with consumer satiation (i.e. seed removal by mice limited by a maximum rate) in years of high seed availability [53], and with the observation that seed predation had no significant effect on the amount of seed available [17].

Our improved resource–consumer models have a number of advantages over previous approaches. First, our consumer model required fewer parameters than other models used with the same data, but had similar or better explanatory power when driven by the appropriate seedfall model (i.e. ΔT; r^2^ = 0·55 cf. 0·223–0·439 in [16]).

Second, a consumer–resource model must correctly predict outbreaks that occurred, and not predict outbreaks at other times [15]. Using the ΔT resource–consumer model, we predicted six of the seven largest outbreaks in the data series (Fig. 1). The exception was the only recorded double peak (in 1974–1975), which none of the models predicted well. Conversely, the model did not predict outbreaks that were not observed in the data. Seasonal fluctuations were evident in the dynamics of our consumer model, consistent with consumer population growth when beech seed was available and with little or no population growth at other times. The magnitude of large outbreaks was underestimated by all the models, similar to results reported by Pech et al. [52], albeit for a very different system. However this might not be critical for using the model to trigger management intervention if damage thresholds are exceeded before peak mouse abundance is reached (cf. [41]). The ΔT-driven model matched the qualitative pattern of outbreaks better than the T-driven model, which failed in particular to predict low periods between outbreaks as well as indicating a lower maximum population level during outbreaks.

Third, our consumer model parameters are explicitly related to demographic processes such as mortality and reproductive rates. This allows comparison of the relative effectiveness of lethal versus fertility control, as has been done for other irruptive species (e.g. [54]). Conversely, other aspects of consumer ecology—such as detailed knowledge of all components of mouse diets—do not need to be modelled explicitly. Some of these additional food types are likely to fluctuate in synchrony with masts (e.g. seed-eating invertebrates). In this sense, the ΔT model is an example of a general approach where climatic variables have been used directly to model the dynamics of irruptive mammalian species, e.g. kangaroos (*Macropus* spp.) [55], foxes (*Vulpes vulpes*) [56], rabbits (*Oryctolagus cuniculus*) [57], feral pigs (*Sus scrofa*) [58] and house mice [15].

Fourth, with the global trend towards collection and modelling of climate data spatially (e.g. [39]), our approach facilitates a much larger spatial scale of prediction for resource pulses and consumer outbreaks than was previously possible. In our mouse–beech system, localized models of beech seed production are needed across the latitudinal and altitudinal range of the South Island when predictions rely on absolute temperatures. However, the responses of *Fuscospora* and other masting species to ΔT are consistent at regional and national scales in New Zealand [25], meaning that large-scale spatial synchrony of resource pulses can be predicted on the basis of regional variation in ΔT. From a management perspective this is extremely useful, since data do not need to be collected at every location for which predictions are required. Our findings may therefore facilitate a better understanding of the mechanisms underlying spatial synchrony in consumer outbreaks worldwide [59–61].

Finally, our improved resource–consumer models allow us to make inference about how changes in resource patterns might affect consumer dynamics in future. This is important when exploring the effects of climate change on resource–consumer dynamics [37, 61]. Increased climatic variability between successive years is a global trend [62]. Although impacts of climate change on variability in ΔT are yet to be formally assessed for all areas of beech forest in New Zealand, our preliminary analysis of the number and distribution of years in which ΔT is greater than some threshold corresponding to a mast event (ΔT_thres_) for three climate scenarios suggests that the effect on mast seeding in the next 100 years at OV may be small. In contrast, using an absolute temperature threshold either predicts increasing temperatures will have major consequences for mast seeding species, or indicates that the T model is not fit for purpose. Our ΔT-driven approach captures climate variability more accurately, with significant implications for resource and consumer dynamics. Moreover, with irruptive consumers, the size of an outbreak is often linked to starting density as well as magnitude of the resource pulse [13, 16, 63]. More frequent resource pulses could enable consumer populations to persist long enough at high density to initiate a subsequent outbreak. In the New Zealand beech–mouse system, successive high-seedfall years (‘double masts’) can have disastrous consequences for native biota [28]. Consequently, any change in climate that alters the frequency of masts, especially double masts, will have flow-on effects for (invasive) consumer outbreaks and subsequent impacts on native biota [37]. Additional research on past occurrences of masts and mouse outbreaks would provide a valuable benchmark for assessing the consequences of climate change for the beech–mouse system.

Our resource–consumer model has some limitations. The density-dependent term for population growth (μ_2_) and the cap on per capita consumption of seed, both necessary to provide a good fit, prevented the model from predicting the exact magnitude of the largest outbreaks. Mouse breeding season (onset and duration) is determined primarily by food supply [17, 41, 64] and the exact timing of the breeding season within a year may vary. This effect was not captured by our consumer model, which assumed that seedfall occurred steadily over 3 months in autumn each year. To improve and parameterize variable timing of the onset and duration of breeding into the model, more frequent collection of seedfall and mouse data would be necessary.

In summary, improved climate-based models as drivers of pulsed resource input to ecosystems improve our ability to predict consumer dynamics, including outbreaks. Because climate information is often collected over large spatial scales, our model has the ability to predict the synchrony that has been observed over large areas for beech masts and house mouse outbreaks in New Zealand. Predictions of resource pulses can be modified to include the increasing variability likely to occur as a result of climate change. The threshold effect is a straightforward and easy-to-use ‘rule of thumb’ for planning: if ΔT < 0, a mast and an outbreak of consumers is unlikely, and therefore no management will be required; however, if ΔT > 1, an outbreak year is almost guaranteed or, if initial density is already high, an outbreak will be maintained. Management strategies can therefore be planned and budgeted for in advance.
